# Advances in Near-Infrared Organic Photodetectors: Molecular Design, Exciton Dynamics, and Device Integration

**DOI:** 10.3390/polym18020201

**Published:** 2026-01-11

**Authors:** Hyosun Lee, Jongho Kim

**Affiliations:** Department of Textile System Engineering, Kyungpook National University, Daegu 41566, Republic of Korea

**Keywords:** near-infrared photodetectors, organic semiconductors, molecular engineering, conjugated polymers, flexible optoelectronics

## Abstract

Near-infrared organic photodetectors (NIR-OPDs) are emerging as versatile platforms for flexible and low-cost optical sensing, yet achieving high-performance in the NIR region remains difficult remains challenging due to intrinsic trade-offs at both the material and device levels, due to the inherent balance required among bandgap narrowing, exciton dissociation, charge transport, and dark-current suppression. This review provides a concise overview of OPD operating mechanisms and the performance metrics governing sensitivity and noise. We highlight recent molecular-engineering strategies—core fluorination, asymmetric π-bridge design, fused-ring rigidification, and polymer backbone/side-chain tuning—that effectively enhance intermolecular ordering, reduce energetic disorder, and extend NIR absorption. Progress in all-polymer detectors and ambipolar phototransistors further demonstrates improved stability and broadened detection capability. Additionally, emerging applications, including NIR communication, biosignal monitoring, flexible imaging, and biometric recognition, showcase the expanding utility of NIR-OPDs. Remaining challenges include pushing detection beyond 1200 nm, simplifying synthesis, and improving long-term stability. Overall, advances in low-bandgap molecular design and device engineering continue to accelerate the practical adoption of NIR-OPDs.

## 1. Introduction

Photodetectors are devices that convert external light signals into electrical signals [1,2,3]. They have become essential components in various optical sensing technologies, including image sensing, optical communication, environmental monitoring, and other related applications [4,5,6,7,8,9]. Traditionally, most commercial photodetectors have been fabricated based on inorganic semiconductors such as silicon and III–V compounds, due to their high carrier mobility, excellent stability, and small exciton binding energy [10,11,12,13]. However, these inorganic photodetectors exhibit several drawbacks, such as rigidity, high manufacturing costs, mechanical fragility, and difficulties in large-area fabrication, which limit the flexibility in both application and design. Furthermore, due to the broadband absorption of inorganic semiconductors, selective detection of specific wavelength regions without external optical filters remains challenging [14,15,16].

In contrast, organic photodetectors (OPDs) offer key advantages such as superior mechanical flexibility, low fabrication costs, and lightweight characteristics [17,18]. These features make them particularly well-suited for applications in flexible, wearable, and portable electronic devices [19,20,21,22]. These favorable characteristics originate from their photoactive layers, which are typically composed of small molecules or polymeric organic semiconductors that contain heteroatoms such as nitrogen, sulfur, and oxygen. The presence of these heteroatoms contributes to weak intermolecular van der Waals interactions, providing mechanical flexibility [17,23]. In addition, their bandgaps can be readily tuned at the molecular level, enabling selective detection in the ultraviolet (UV), visible, and near-infrared (NIR) regions without the need for external filters [22,24,25,26].

In particular, OPDs operating in the NIR (λ = 700–1000 nm) region can penetrate biological tissues more deeply than visible light and are safer for human exposure compared to UV or visible wavelengths, while generating less heat upon absorption [27,28,29,30]. Therefore, NIR OPDs are highly suitable for sensitive biological and industrial applications in various fields, including bioelectronics, medical diagnostics, night vision, and facial recognition [31,32].

However, the performance of current NIR-OPDs still lags behind that of UV-Vis OPDs. This is mainly due to several intrinsic material and device-level limitations:

- Designing materials for NIR absorption is challenging, as it requires balancing strong NIR absorption with high charge carrier mobility and efficient exciton dissociation.
- The excitonic nature of conjugated polymers leads to tightly bound Frenkel excitons, which are difficult to dissociate. This hinders charge separation, increases recombination, and ultimately lowers device efficiency [33,34].
- Narrow highest occupied molecular orbital (HOMO)—lowest unoccupied molecular orbital (LUMO) gap lowers the injection barrier at the electrode interface under dark conditions, leading to increased dark current [35].

To overcome these issues, numerous scientists have focused on developing materials and optimizing device architectures to achieve low dark current, high external quantum efficiency (EQE), and fast response speeds simultaneously [36,37].

**Figure 1:**
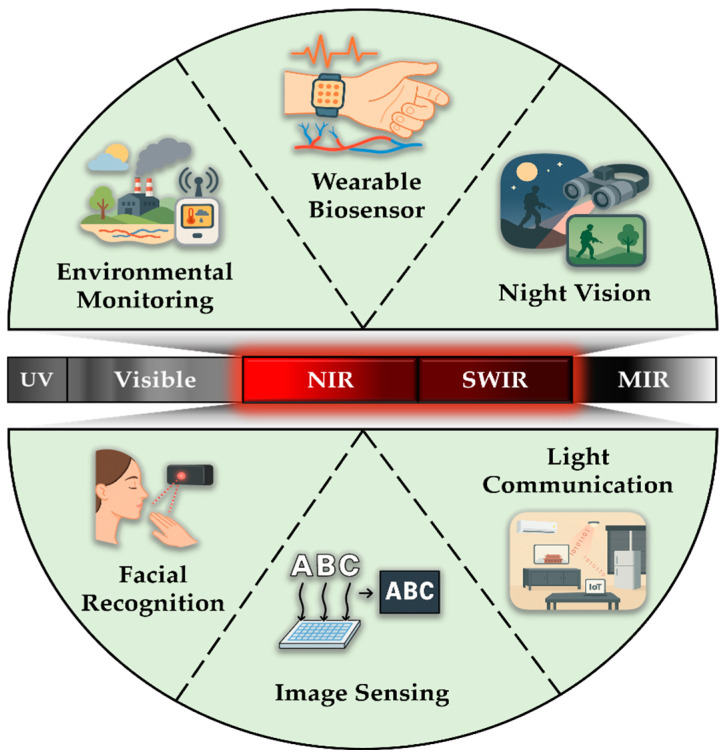
Applications of NIR OPDs: wearable biosensors, night vision, light communication, image sensing, facial recognition, and environmental monitoring.

Accordingly, this review outlines the fundamental principles and key parameters of photodetectors, followed by recent research trends in material selection and device performance optimization for NIR OPDs. It also introduces practical application areas of NIR OPDs and discusses future directions for their development (Figure 1).

## 2. Fundamental Theory of Organic Photodetectors

### 2.1. Mechanism of Organic Photodetectors

**Figure 2:**
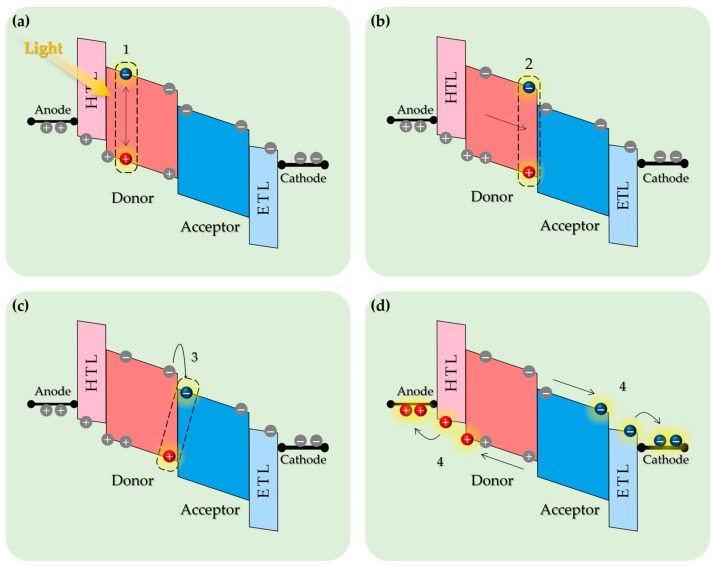
Illustration of the working mechanism of an organic photodetector: (**a**) Exciton generation. (**b**) Exciton diffusion. (**c**) Charge separation. (**d**) Charge transport and collection.

The working principle of OPDs is based on the internal photoelectric effect. As shown in Figure 2, the process by which optical signals are converted into electrical signals in OPDs can be explained in four steps:

- Photon absorption and exciton generation (Figure 2a): When photons are absorbed by the active layer, electrons in the HOMO are excited to the LUMO, generating excitons. In inorganic semiconductors, these excitons (often Wannier–Mott type) exhibit very low binding energies, typically around ~1 meV, allowing for easy dissociation [38,39]. Conversely, excitons in organic materials are typically of the Frenkel type, with significantly higher binding energies (~1 eV), making their spontaneous dissociation difficult [40,41].
- Exciton diffusion (Figure 2b): The generated excitons are spatially confined within individual molecules, but they can diffuse toward the donor–acceptor interface via Förster (through-space) and Dexter (through-bond) energy transfer mechanisms. However, the short exciton diffusion length characteristics of organic semiconductors often limit their ability to reach the interface [42,43].
- Interfacial charge dissociation (Figure 2c): At the donor–acceptor interface, an energy offset arising from differences in ionization energy and electron affinity facilitates the dissociation of the excitons into charge transfer states (CTSs), which subsequently separate into free electrons and holes. An energy offset of approximately 0.3 eV is generally required to efficiently facilitate charge separation [44,45]. The separated CTSs can diffuse over distances of 5–10 nm along the interface; however, if they do not fully dissociate into free charges, they recombine back to the ground state via geminate recombination [46,47,48].
- Charge carrier transport and collection (Figure 2d): The separated free electrons and holes migrate to their respective electrodes for collection. Although organic semiconductors typically exhibit lower charge carrier mobility than their inorganic counterparts—potentially limiting the response speed—this limitation can be reduced by optimizing the active layer morphology and applying an external electric field [49,50].

### 2.2. Key Performance Parameters of Organic Photodetectors

#### 2.2.1. External Quantum Efficiency (EQE) and Responsivity (*R*)

EQE is a key parameter that reflects the photoelectric conversion performance of OPDs [51,52]. It represents how many incident photons at a specific wavelength are effectively converted into charge carriers and subsequently collected by the electrodes. EQE is typically expressed as a percentage; a higher EQE indicates greater efficiency in charge generation per incident photon.

(1) $$\mathrm{EQE}=\frac{number\;of\;collected\;charge\;carriers}{number\;of\;incident\;photons}=\frac{\frac{I_{ph}}{q}}{\frac{P_{in}}{h\nu }}=\frac{I_{ph}h\nu }{P_{in}q}=\frac{I_{ph}hc}{P_{in}q\lambda }=R\frac{hc}{q\lambda }=R\frac{1240}{\lambda }\times 100[\%]$$

Here, *I_ph_* is the photocurrent, *P_in_* is the incident light power, *λ* is the wavelength of the incident light, *h* is Planck’s constant, *c* is the speed of light, *q* is the elementary charge, and *ν* is the frequency of the incident light.

Responsivity (*R*) is defined as the ratio of the generated photocurrent to the incident optical power at a specific wavelength, with units of A/W [53].

(2) $$R=\frac{I_{ph}}{P_{in}}\;=\mathrm{EQE}\frac{q\lambda }{hc}\;[A/W]$$

According to Equation (2), responsivity can be expressed as *R* = *I_ph_*/*P_in_* and is directly related to EQE at the same wavelength λ through the factor *qλ*/*hc*. Therefore, when *I_ph_* and *P_in_* are measured under the same illumination conditions, EQE and *R* provide a consistent description of the photoelectric conversion performance of OPDs at that wavelength.

#### 2.2.2. Dark Current and Noise

In photodetectors, dark current and various intrinsic electronic noise sources critically influence the device’s signal detection performance [54,55,56]. Representative noise types include shot noise, thermal noise, flicker noise (1/f noise), and generation–recombination (G–R) noise [57,58,59].

- Dark Current

Dark current is the small leakage current that flows under reverse bias in the absence of light. It primarily arises from thermally generated carriers, defect-assisted leakage, and interfacial recombination. Since dark current directly contributes to the total noise of the device, its effective suppression is highly important. Common strategies for reducing dark current include device cooling, interfacial passivation, and optimization of the reverse bias voltage.

- Shot Noise

Shot noise originates from the discrete nature of electric charge and represents one of the fundamental noise sources in photodetectors. It is determined by the total current, which includes the signal photocurrent, background-induced photocurrent, and dark current:

(3) $$i_{\mathrm{shot}}=i_{\mathrm{signal}}+i_{\mathrm{background}}+i_{\mathrm{dark}}\approx i_{\mathrm{dark}}=\sqrt{2qI_{d}\Delta f}$$

Here, *q* is the elementary charge, *i_signal_* is the photocurrent generated by incident light, *i_background_* arises from background radiation, *i_dark_* is the dark current, and *Δf* is the noise bandwidth. In the shot noise formulation, the dark current is denoted as *I_d_*. Under typical laboratory conditions, *i_signal_* and *i_background_* are often negligible compared to *i_dark_*, making dark current the dominant source of shot noise.

- Thermal Noise

Thermal noise (also known as Johnson–Nyquist noise) arises from the random thermal motion of charge carriers and exists even without an applied bias. It mainly occurs in resistive components and is caused by voltage fluctuations due to thermal energy. It is commonly observed in narrow-bandgap materials used for NIR detection. The thermal noise current is given by:

(4) $$i_{\mathrm{thermal}}\;=\;\sqrt{\frac{4k_{B}T\Delta f}{R_{shunt}}}$$

where *k_B_* is Boltzmann’s constant, *T* is the absolute temperature, and *R_shunt_* is the shunt resistance.

- Other Noise Sources

Additionally, flicker noise (1/f noise), which is significant at low frequencies, is often associated with charge trapping and detrapping dynamics. Generation–recombination (G–R) noise arises from carrier transitions involving trap states. These noise components are more complex and cannot be fully described by simple device parameters.

- Total Noise Current

Since the noise sources act independently, the total noise current (*i_noise_*) can be approximated as the square root of the sum of their individual contributions squared:

(5) $$i_{\mathrm{noise}}=\;\sqrt{{i_{shot}}^{2}+{i_{thermal}}^{2}+{i_{1/f}}^{2}+{i_{G-R}}^{2}}$$

#### 2.2.3. Signal-to-Noise Ratio (SNR)

The signal-to-noise ratio (SNR) refers to the ratio of the signal current to the noise current of a photodetector. The SNR quantitatively evaluates the device’s ability to distinguish valid signals from background noise. Achieving a high SNR is essential, as it indicates that the detector can identify signals more clearly and sensitively in noisy environments.

(6) $$\mathrm{SNR}=\frac{I_{ph}}{i_{noise}}$$

#### 2.2.4. Noise Equivalent Power (NEP)

The noise equivalent power (NEP) is defined as the minimum optical power required for a detector to produce a signal equal to the noise level within a 1 Hz bandwidth. It represents the lowest detectable power of the photodetector. The unit is W·Hz^−1/2^.

(7) $$\mathrm{NEP}=\frac{I_{ph}}{R}=\frac{i_{noise}}{R}\left[W/\sqrt{Hz}\right]$$

#### 2.2.5. Specific Detectivity (D*)

Specific detectivity (D*) quantifies the sensitivity and signal discrimination capability of a detector, allowing for direct comparison across devices with different sizes and noise characteristics. It indicates how well the device can distinguish weak optical signals from noise. The unit is Jones (cm·Hz^1/2^/W).

(8) $$D*=\frac{\sqrt{A\Delta f}}{NEP}=\frac{R\sqrt{A}}{i_{noise}}\left[cm\sqrt{Hz}/W\right]$$

As shown in Equation (8), D* is defined from the noise equivalent power (NEP) and can be expressed in terms of responsivity (*R*) and total noise current (*i_noise_*). Here, *A* denotes the effective active area of the photodetector, which enables a fair comparison of detection performance across devices with different sizes. Therefore, specific detectivity reflects the combined influence of responsivity and noise and serves as a comprehensive figure of merit for evaluating the sensitivity of organic photodetectors.

#### 2.2.6. Linear Dynamic Range (LDR)

The linear dynamic range (LDR) defines the range of incident optical powers over which the detector’s output response remains linear. It is typically expressed in decibels (dB) and is calculated as follows:

(9) $$\mathrm{LDR}\;=\;20\log_{10}(\frac{I_{max}}{I_{min}})\;[dB]$$

where *I_max_* and *I_min_* represent the maximum and minimum detectable currents within the linear regime. A larger LDR indicates that the detector can linearly respond to a wider range of light intensities.

#### 2.2.7. Frequency Response

The frequency characteristics of OPDs are commonly described by the frequency response of the photocurrent to a modulated optical signal, where the light intensity is intentionally varied with time to probe the temporal response of the device. As the modulation frequency increases, the photocurrent amplitude decreases because charge generation, transport, and extraction processes cannot fully follow rapid variations in the incident light. A representative metric is the −3 dB cutoff frequency, defined as the frequency at which the response decreases to 1/$\sqrt{2}$ of its low-frequency value, and it is widely used to indicate the maximum operating speed of a photodetector. These characteristics are governed by carrier transit, trapping–detrapping dynamics, and device RC time constants, and therefore should be considered together with responsivity and noise for application-specific performance evaluation [60,61].

#### 2.2.8. Selectivity in NIR OPDs

Selectivity in near-infrared organic photodetectors depends on the intended application. In applications such as biomedical imaging and environmental sensing, selective detection of NIR light with suppressed visible-light response is required [62,63]. In contrast, applications such as optical communication, multispectral imaging, and organic solar cells benefit from broadband photodetection extending from the visible to the NIR and even the short-wave infrared (SWIR) region [64,65]. Accordingly, NIR OPDs can be classified as NIR-selective or NIR-extended broadband devices, and their design strategies and performance metrics should be considered in relation to the target application.

## 3. Organic Semiconductor Materials for NIR OPDs

In OPDs, organic semiconductors are generally classified into small molecules and polymers, which share common design principles such as π-conjugated backbones and donor–acceptor motifs for efficient light absorption and charge transport. Despite these similarities, fundamental differences in molecular architecture lead to distinct structure–performance relationships.

Small-molecule semiconductors possess discrete molecular structures with well-defined molecular weights, enabling high purity, excellent reproducibility, and precise control over energy levels and optical absorption [66,67]. Their rigid backbones and ordered intermolecular packing can result in high charge carrier mobility and sharp absorption features under favorable crystallinity [68]. However, excessive crystallization often causes morphological instability, phase separation, and reduced mechanical robustness, while thin-film formation remains highly sensitive to processing conditions [69,70,71].

In contrast, polymer semiconductors consist of repeating conjugated units forming long-chain architectures with distributed molecular weights. This intrinsic chain connectivity provides excellent solution processability and mechanical flexibility, making polymers attractive for low-cost, large-area, and flexible OPD applications [72,73]. In addition, energy levels and optical properties can be systematically tuned through backbone engineering and side-chain modification [74,75,76]. However, the presence of structural disorder and inter-chain charge transport typically leads to lower charge carrier mobility compared to highly ordered small-molecule systems. Moreover, variations in molecular weight distribution and processing conditions complicate precise control of film morphology and limit morphological reproducibility [72,77].

Owing to these complementary characteristics, both small molecules and polymers serve as essential material platforms for high-performance organic photodetectors.

### 3.1. Small-Molecule Materials

**Figure 3:**
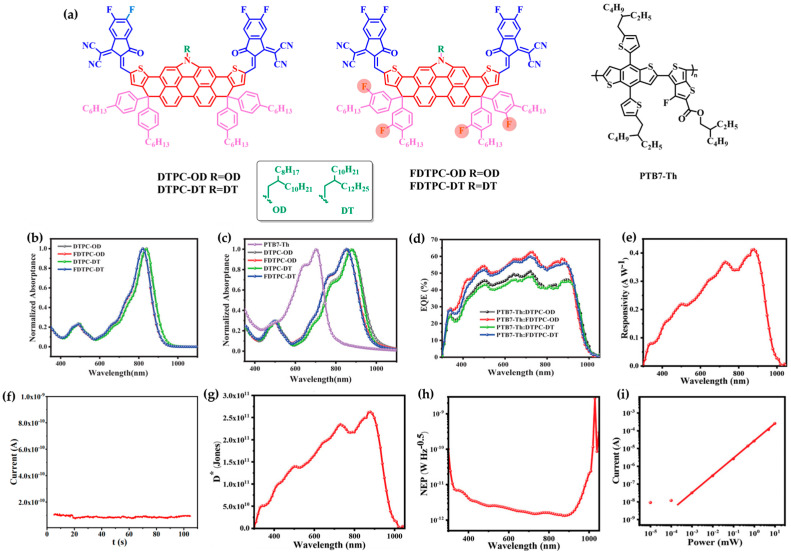
(**a**) Chemical structures of DTPC-OD, DTPC-DT, FDTPC-OD, FDTPC-DT and PTB7-Th. (**b**) UV–Vis absorption spectra of DTPC-OD, FDTPC-OD, DTPC-DT, and FDTPC-DT in dichloromethane solution. (**c**) Thin-film absorption spectra of PTB7-Th, DTPC-OD, FDTPC-OD, DTPC-DT, and FDTPC-DT. (**d**) EQE spectra of PTB7-Th:acceptor blend devices. (**e**) Responsivity of PTB7-Th:FDTPC-OD-based device. (**f**) Dark current of PTB7-Th:FDTPC-OD device at a bias voltage of −0.1 mV. (**g**) NEP and (**h**) Detectivity of PTB7-Th: FDTPC-OD. (**i**) Photocurrent versus light power curves of PTB7-Th: FDTPC-OD. Reproduced with permission from Ref. [78]. Copyright 2022, Wiley-VCH GmbH.

In 2022, Chen’s group developed a high-performance NIR photodetector based on FDTPC-OD, a narrow-bandgap small-molecule acceptor featuring a 2D fluorinated central core [78]. The molecule adopted an acceptor–donor–acceptor (A–D–A) configuration, consisting of a dithienopyrrole-carbazole (DTPC) electron-donating core and electron-withdrawing 2-(5,6-difluoro-3-oxo-2,3-dihydro-1H-inden-1-ylidene)malononitrile (DFIC) terminal groups (Figure 3a). This structure facilitated strong intramolecular charge transfer (ICT), enabling NIR absorption. The incorporation of fluorine atoms into the core effectively modulated energy levels and enhanced molecular packing, which promoted efficient charge generation and separation. Moreover, tuning the alkyl chain length on the nitrogen of the pyrrole unit significantly influenced molecular ordering and optoelectronic properties; longer chains led to increased disorder and degraded device performance.

FDTPC-OD exhibited an absorption onset extending to 851 nm in thin films (Figure 3b,c), corresponding to an optical bandgap of 1.26 eV, characteristic of typical NIR absorbers. Devices based on PTB7-Th:FDTPC-OD demonstrated EQE in the NIR region, with a *R* exceeding 0.4 A/W at 880 nm, a low dark current of 8 × 10^−11^ A, and a D* above 2.5 × 10^11^ Jones (Figure 3d–g). Additionally, the device achieved a NEP of 1.3 × 10^−12^ W Hz^−1/2^ and a LDR of 78.1 dB (Figure 3h,i). Furthermore, the device showed balanced charge transport, rapid charge separation, long carrier lifetime, and high exciton dissociation efficiency, indicating favorable overall charge dynamics.

This work provides compelling evidence that core fluorination and precise control of alkyl substitution can be effective strategies for simultaneously optimizing energy levels, molecular packing, and photoresponse characteristics in A–D–A small-molecule acceptors for NIR OPDs.

**Figure 4:**
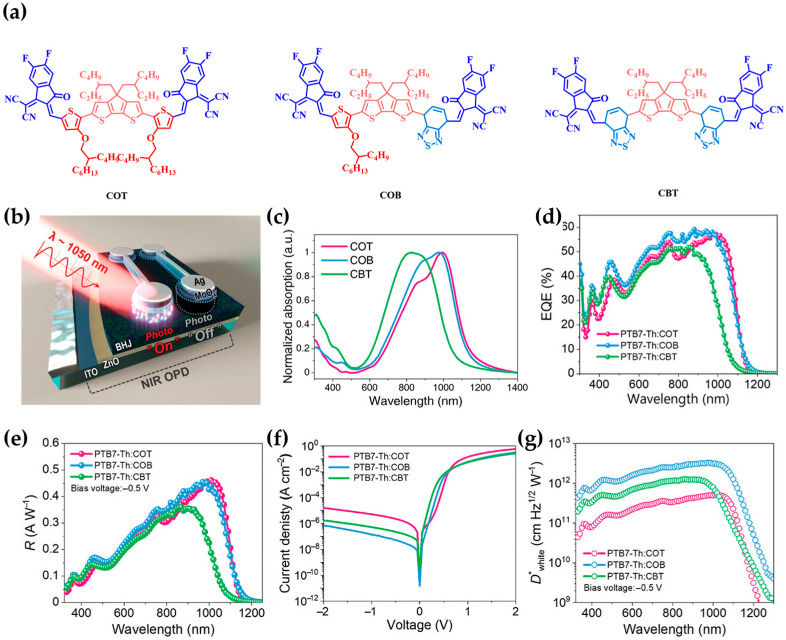
(**a**) Chemical structures of COT, COB, and CBT. (**b**) Normalized absorption spectra of their thin films. (**c**) Schematic of the OPD device architecture. (**d**) EQE spectra of the OPD devices measured under AM 1.5 G illumination (100 mW/cm^2^). (**e**) Specific responsivity at −0.5 V. (**f**) J–V characteristics of the OPD devices under dark conditions. (**g**) White-noise-limited specific detectivity (D*) at 1050 nm, under a bias of −0.5 V. Reproduced with permission from Ref. [79]. Copyright 2023, Wiley-VCH GmbH.

In 2023, a research team including Ko reported a high-sensitivity NIR OPD based on a narrow-bandgap asymmetric acceptor named COB, designed through π-bridge engineering [79]. The molecule adopted an A–π–D–π–A architecture incorporating an electron-rich central donor cyclopentadithiophene (CPDT) and strongly electron-deficient IC-2F terminal groups. Importantly, these A–π–D–π–A structures incorporated asymmetrically positioned π-bridges—alkoxythienyl (OT) or benzothiadiazole (BT)—with distinct electronic properties (Figure 4a).

By introducing the strongly electron-deficient BT unit, the molecule retained a strong ICT character while lowering the HOMO level to –5.30 eV, thereby achieving both broad absorption and excellent photoresponse characteristics. In the solid state, COB exhibits a maximum absorption peak near 980 nm and an absorption edge extending to ~1140 nm, corresponding to an optical bandgap of 1.08 eV, favorable for NIR detection (Figure 4b).

Notably, the asymmetric π-bridge enabled a balanced push–pull effect and moderate solubility. This balance suppressed the excessive aggregation typically observed in low-solubility systems. As a result, COB achieved a loosely packed stacking arrangement and favorable phase blending, which reduced trap densities and facilitated efficient charge separation and transport. This contributed significantly to the overall enhancement of the device’s photodetection performance.

Accordingly, devices based on PTB7-Th: COB blends (Figure 4c) demonstrated high EQE over the 300–970 nm range and maintained efficient photoresponse even beyond 1050 nm. The optimized device also exhibited an *R* of 0.369 A/W, a low dark current of 5.22 × 10^−8^ A/cm^2^, and a D* of 2.68 × 10^12^ Jones under white-noise-limited conditions (primarily comprising shot and thermal noise) with a −0.5 V bias at 1050 nm (Figure 4c–f).

These findings highlight that asymmetrically tuning the electronic properties of π-bridges within the A–π–D–π–A framework provides a compelling design strategy for simultaneously optimizing ICT strength, energy level alignment, and molecular packing.

To enhance the performance of OPDs operating in the NIR region, the development of materials with optimized optoelectronic characteristics is crucial. One effective strategy to achieve these characteristics is employing all-fused-ring n-type small molecules. These molecules feature rigid and planar π-conjugated backbones that minimize conformational disorder, thereby reducing trap states and dark current. However, despite these advantages, the synthesis of fully fused-ring systems remains challenging, and only a few such materials have been reported [80,81,82,83].

**Figure 5:**
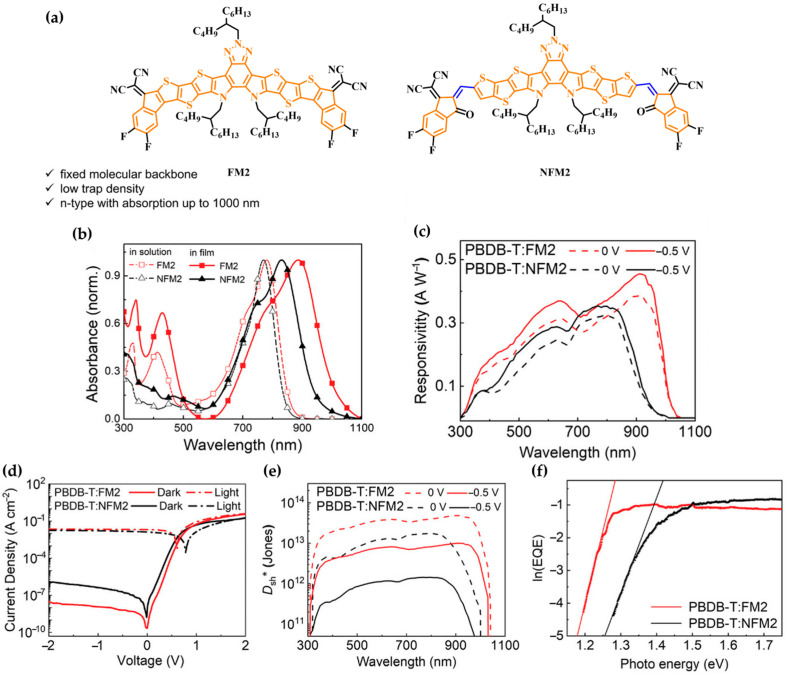
(**a**) Chemical structures of FM2 with an all-fused-ring backbone and NFM2 with a partially fused-ring structure. (**b**) Absorption spectra of FM2 and NFM2 in solution and in the thin film. (**c**) Responsivity spectra at 0 and −0.5 V. (**d**) Current density–voltage (J–V) characteristics under dark and illuminated conditions. (**e**) D_sh_* at 0 and −0.5 V for the optimized OPD devices based on PBDB-T:FM2 and PBDB-T: NFM2. (**f**) Extraction of Urbach energy from ln (EQE) in the long-wavelength edge. Reproduced with permission from Ref. [84]. Copyright 2023, Wiley-VCH GmbH.

To address these limitations, Jun Liu and co-workers in 2023 developed an all-fused-ring n-type acceptor molecule, FM2, comprising 14 fused π-conjugated rings [84]. This molecule enabled high-sensitivity NIR detection beyond 1000 nm. FM2 adopts an ADA’DA structure and consists of an electron-deficient benzotriazole core, two electron-rich thieno(2″″,3″″:4″,5″]thieno(2″,3″:4,5]thieno(3,2-b]pyrrole bridging units, and two malononitrile-functionalized electron-deficient terminals (Figure 5a). This molecule promotes strong ICT, resulting in high structural rigidity and a low Stokes shift. While inspired by the partially fused architecture of Y6-type acceptors, FM2 adopts an all-fused-ring configuration with an extended π-conjugated system and a narrower bandgap, leading to significantly enhanced NIR absorption.

Compared to the partially fused NFM2, the all-fused FM2 exhibited a more pronounced red-shift (106 nm) in film due to stronger intermolecular interactions, resulting in a lower optical bandgap of 1.22 eV and extended NIR absorption up to 1013 nm—favorable for long-wavelength photodetection (Figure 5b). Inverted OPDs based on PBDB-T:FM2 blends achieved an *R* of 0.372 A/W at 880 nm, which increased to 0.455 A/W under −0.5 V bias. The devices also exhibited a low dark current of 2.01 × 10^−10^ A/cm^2^ and a shot-noise-limited D_sh_* of 4.65 × 10^13^ Jones (Figure 5c–e). The reduced energetic disorder of FM2, evidenced by a lower Urbach energy (*E_U_*) of 22.3 meV compared to 34.2 meV for NFM2 (Figure 5f), contributed to suppressed trap-assisted recombination and thus enabled high responsivity and fast photoresponse through more efficient carrier extraction.

This work demonstrates that the rigid design of all-fused-ring acceptors can be a highly effective approach for reducing trap density and suppressing dark current, thereby improving OPD performance in the NIR region.

In 2022, Wang’s group and their coworkers reported high-performance NIR OPDs based on a non-fused A–D–A′–D–A-type acceptor, TPDC-4F. Unlike the previously described fused-ring ADA′DA structures, the non-fused TPDC-4F offers greater structural flexibility while significantly reducing synthetic complexity and cost [85]. This enhanced design freedom enabled more precise tuning of optical absorption and electronic structure, resulting in broad spectral coverage and low-bandgap characteristics.

**Figure 6:**
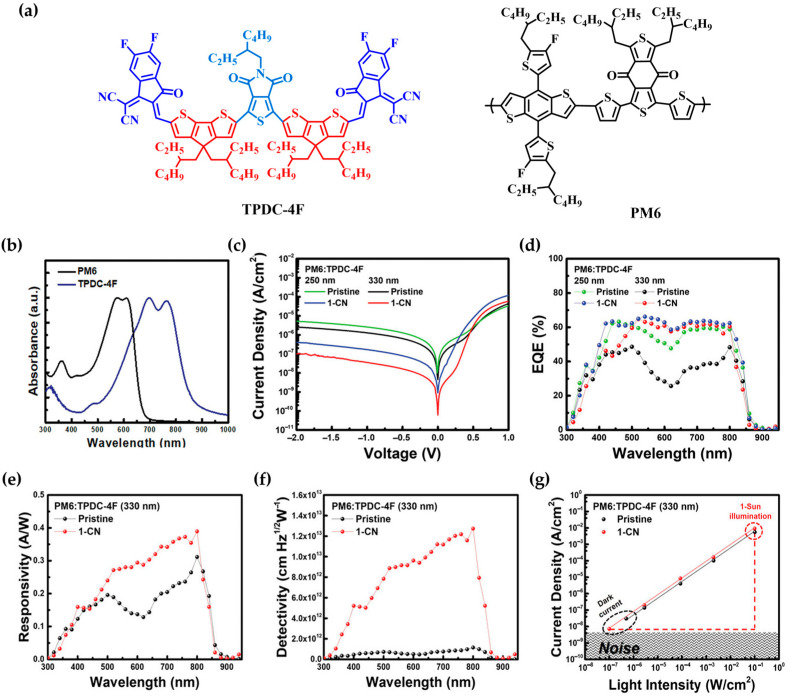
(**a**) Molecular structures of PM6 and TPDC-4F. (**b**) UV–vis absorption spectra of PM6 and TPDC-4F in the thin-film state. (**c**) J–V characteristics of the OPD under dark conditions. (**d**) EQE spectra of PM6: TPDC-4F-based OPDs with varying active layer thickness and additive content (applied bias: −0.1 V). (**e**) Wavelength-dependent responsivity. (**f**) Specific detectivity calculated from the dark current density. (**g**) LDR of PM6: TPDC-4F-based OPDs with and without 1-CN additive (applied bias: −0.1 V). Reproduced with permission from Ref. [85]. Copyright 2022, Wiley-VCH GmbH.

The TPDC-4F incorporates an electron-deficient diketone core with pronounced quinoidal character, thereby enhancing π-electron delocalization. Furthermore, intramolecular O···S noncovalent interactions between the core, bridging, and terminal units improve molecular planarity and facilitate ICT (Figure 6a).

In thin-film form, TPDC-4F exhibits a maximum absorption peak around 760 nm, with NIR absorption extending to 850 nm. Its optical bandgap was determined to be 1.42 eV (Figure 6b). The active layer was composed of the donor polymer PM6 and TPDC-4F, with vertical phase separation induced by the use of 1-chloronaphthalene (1-CN) as an additive. Due to the surface energy difference, TPDC-4F exhibits a stronger affinity to 1-CN than PM6. As a result, TPDC-4F preferentially migrated to the top surface during film formation with 1-CN, inducing vertical phase separation. This morphology enhanced electron transport and effectively suppressed dark current.

Devices based on the PM6:TPDC-4F blend exhibited a low dark current density of 3.55 × 10^−9^ A cm^−2^, a broad EQE response across 300–900 nm with a peak EQE of ≈80%, and *R* of 0.39 A W^−1^ at 800 nm. The D* was measured to be 1.27 × 10^13^ Jones, and the device demonstrated a wide LDR of 122 dB under an applied bias of −0.1 V (Figure 6d–g).

This study demonstrates that high-performance OPDs can be achieved using structurally simple acceptor material combined with additive-assisted morphological optimization, achieving performance comparable to fused-ring acceptors by leveraging effective interfacial control.

### 3.2. Polymeric Materials

In 2022, the group led by Leem [86] developed high-performance short-wave infrared (SWIR, λ = 1000–2500 nm) OPDs by designing a donor–acceptor copolymer incorporating a thiadiazolo [3,4-g]quinoxaline (TQ)-based core, known for its strong electron-accepting character. In this study, tri-thiophene units were employed as the donor segment in the P1 polymer, and a series of alkoxy substituents were introduced to finely tune the optical absorption and device performance (Figure 6a). As a result, the polymer film exhibited an extended absorption up to 1160 nm and a narrow optical bandgap of approximately 0.83 eV, effectively covering the entire SWIR region (Figure 6b).

The P1 polymer demonstrated favorable molecular ordering with a predominant face-on orientation, attributed to the extended conjugation and linear geometry of the tri-thiophene donor unit, in contrast to the P2 counterpart. Furthermore, the length of the alkoxy side chains significantly influenced the film morphology, crystallinity, and blend miscibility. Among them, the P1 variant with intermediate side chain length yielded a homogeneous blend morphology and enhanced face-on stacking, leading to efficient charge transport, suppressed noise, and improved device performance.

**Figure 7:**
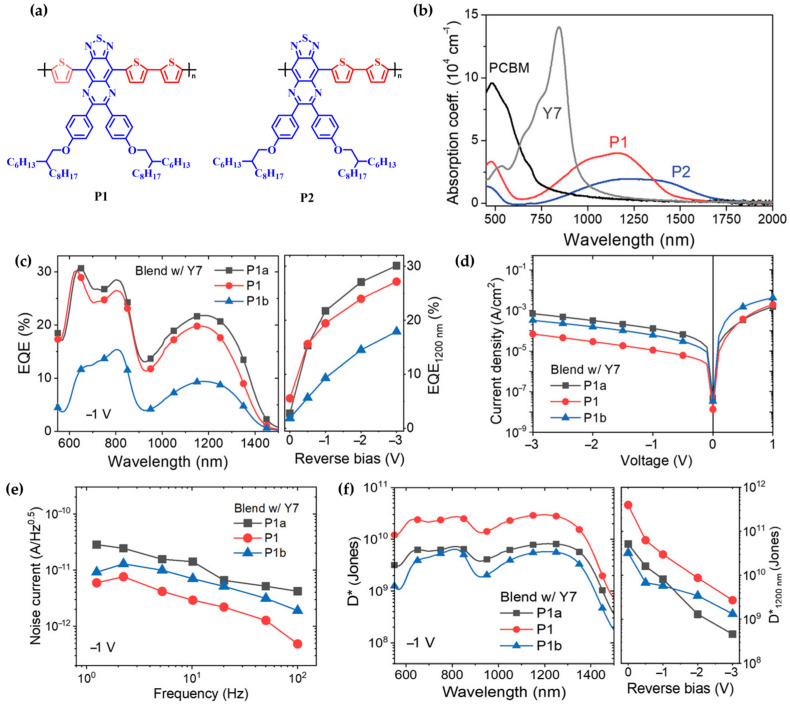
(**a**) Molecular structures of P1 and P2 polymers. (**b**) UV–vis absorption spectra of P1 and P2 polymers. (**c**) EQE spectra measured at −1 V. (**d**) J–V characteristics of OPDs measured in the dark. (**e**) Noise current density as a function of frequency at −1 V. (**f**) Wavelength-dependent specific detectivity at −1 V (**left**) and bias-dependent D* values at 1200 nm (**right**). Reproduced with permission from Ref. [86]. Copyright 2022, Wiley-VCH GmbH.

OPDs fabricated using P1 blended with the nonfullerene acceptor Y7 achieved EQEs of 19.5–21.7% at 1200 nm under −1 V bias. The devices exhibited a low dark current of 1.12 × 10^−5^ A cm^−2^ and, owing to their low noise characteristics, a high D* of 2.96 × 10^10^ Jones (Figure 7c–f). Additionally, fast photoresponse characteristics were observed at 1200 nm, indicating stable operation under real-world conditions.

These findings highlight that copolymer design based on TQ cores, combined with precise side-chain engineering, serves as an effective strategy to achieve both high sensitivity and rapid response in the SWIR detection range.

**Figure 8:**
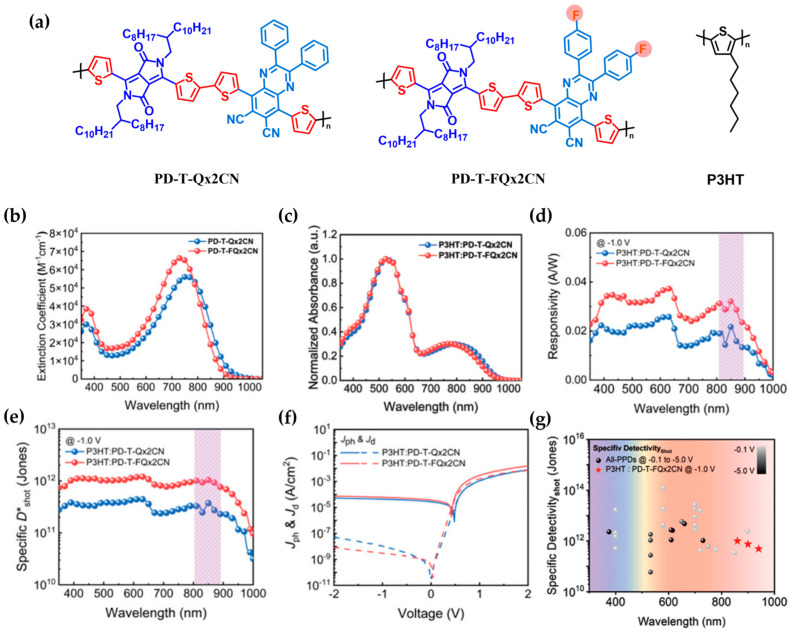
(**a**) Chemical structures of the donor polymer P3HT and the acceptor polymers PD-T-Qx2CN and PD-T-FQx2CN. (**b**) UV–vis absorption spectra of the acceptor polymers measured in CHCl_3_ solution. (**c**) Normalized absorption spectra of the donor–acceptor polymer blend films. (**d**) Responsivity of the P3HT: acceptor blend devices measured at −1.0 V. (**e**) Specific detectivity (D*_sh_) of the corresponding devices. (**f**) Current density–voltage (J–V) characteristics measured under 860 nm illumination and in the dark. (**g**) Comparison of specific detectivity with previously reported all-polymer photodetectors. Reproduced with permission from Ref. [87]. Copyright 2024, Wiley-VCH GmbH.

In 2024, the research group led by Kim [87] developed two n-type polymer acceptors, PD-T-Qx2CN and PD-T-FQx2CN, based on an A1–π–A2 architecture, and employed them as active-layer materials in all-polymer NIR photodetectors via 2:1 (*w*/*w*) blending with P3HT (Figure 8a). Both acceptors consist of an electron-deficient diketopyrrolopyrrole (DPP, A1) unit and a dicyano-substituted quinoxaline (A2) unit, linked by a dithiophene π-bridge. In PD-T-FQx2CN, fluorine atoms were introduced at the 2,3-positions of the benzene ring in the Qx unit to modulate the electronic structure and enhance backbone regularity. This structural modification affected the optical absorption characteristics, energy levels, and film crystallinity.

Figure 8b,c present the absorption spectra of PD-T-Qx2CN and PD-T-FQx2CN measured in chloroform solution and in blended films with the P3HT donor. In the solid state, PD-T-Qx2CN and PD-T-FQx2CN exhibited NIR absorption (700–900 nm), with maximum peaks at 795 nm and 782 nm, respectively. Under −1.0 V bias at 860 nm, responsivity values of 0.020 A W^−1^ (Qx-PD) and 0.031 A W^−1^ (FQx-PD) were observed. Corresponding shot-noise-limited specific detectivities (D*_sh_) were 3.39 × 10^11^ Jones and 1.01 × 10^12^ Jones, respectively, primarily attributed to the lower dark current density of FQx-PD (2.99 × 10^−9^ A cm^−2^) compared to Qx-PD (1.06 × 10^−8^ A cm^−2^) at −1 V (Figure 8d–f).

Moreover, additional device parameters—including NEP, −3 dB cut-off frequency, LDR, and Urbach energy—supported the superior device stability and sensitivity of FQx-PD, driven by faster photoresponse, broader operational range, and reduced energetic disorder.

These findings highlight the effectiveness of fluorination in tuning the electronic structure and film morphology of polymer backbones. The PD-T-FQx2CN-based device demonstrated significantly enhanced detectivity in the NIR range compared to conventional all-polymer photodetectors (Figure 8g). This strategic molecular design provides a promising approach for the development of high-performance A1–π–A2-type polymer acceptors applicable to advanced NIR photodetection technologies.

**Figure 9:**
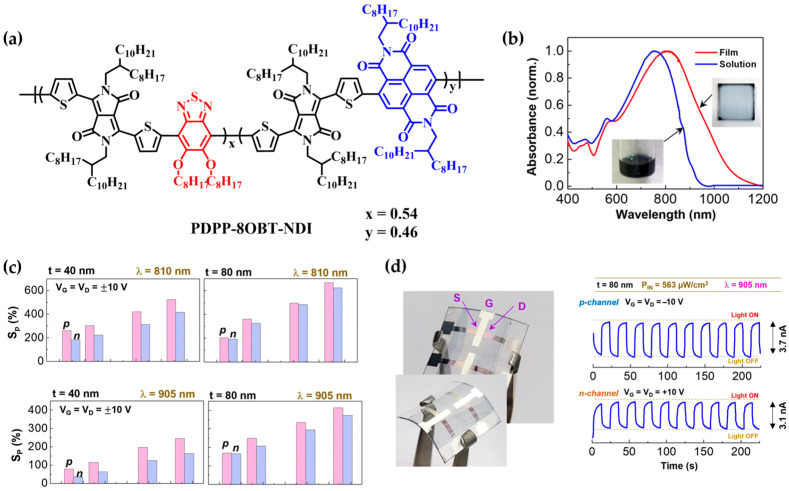
(**a**) Chemical structure of PDPP-8OBT-NDI. (**b**) Optical absorption spectra of PDPP-8OBT-NDI in toluene solution (blue line) and as a 20 nm thick thin film (red line). (**c**) Variation in photosensitivity (*S_P_*) of OPTR devices with 40 nm and 80 nm thick PDPP-8OBT-NDI channels as a function of NIR light intensity (*P_IN_*) under V_G_ = V_D_ = ±10 V. (**d**) Flexible NIR-OPTR device incorporating an 80 nm thick PDPP-8OBT-NDI channel: (**left**) photograph of the bent device; (**right**) photocurrent response under repeated on/off modulation of NIR illumination (λ = 905 nm). Reproduced with permission from Ref. [89]. Copyright 2023, The Royal Society of Chemistry.

More recently, beyond conventional unipolar architectures, ambipolar organic phototransistors (OPTRs) capable of both p- and n-type operation have been actively explored for NIR OPD applications [88]. In 2023, Kim and colleagues [89] developed a high-performance NIR-OPTR by synthesizing a conjugated terpolymer composed of diketopyrrolopyrrole (DPP), benzothiadiazole (BT), and naphthalene diimide (NDI), which exhibited broad NIR absorption and ambipolar charge transport characteristics (Figure 9a).

The device demonstrated a wide absorption range up to 1100 nm and showed photosensitivity values of 622–665% at 810 and 905 nm (Figure 9b), along with stable on/off photoresponses. As the NIR light intensity increased, the photosensitivity (*S_p_*) of the NIR-OFETs gradually increased for both wavelengths, which is attributed to the enhanced drain current under illumination. Notably, the *S_p_* values were consistently higher in p-channel operation than in n-channel operation (Figure 9c). Moreover, OPTR devices fabricated on flexible PEN substrates maintained reliable photocurrent responses under repeated NIR illumination cycles (Figure 9d).

These results suggest that the PDPP-8OBT-NDI-based OPTR, with its broad NIR absorption and excellent ambipolar detection characteristics, holds strong potential for future applications in flexible optical sensors, including laser imaging detection and ranging (LiDAR) systems.

## 4. Applications of Near-Infrared Organic Photodetectors

**Figure 10:**
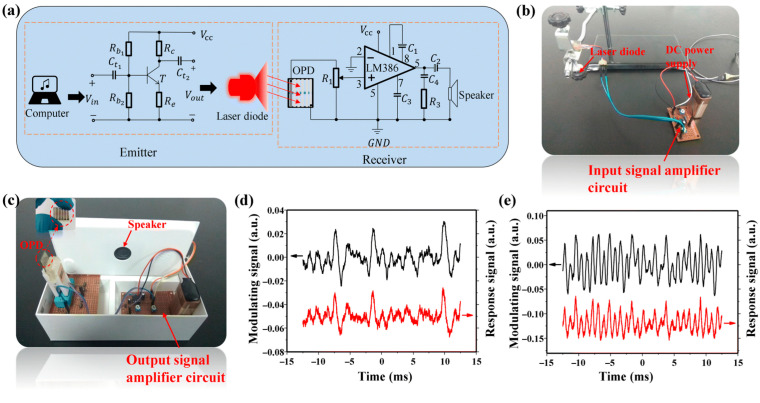
(**a**) Circuit diagram of the laser microphone system. (**b**) Signal transmission unit. (**c**) Signal reception unit. (**d**) Waveform diagrams of low-frequency and (**e**) high-frequency input (black line) and output (red line) audio signals used for NIR optical communication. Reproduced with permission from Ref. [90]. Copyright 2023, The Royal Society of Chemistry.

Various applications have demonstrated the use of OPDs. One notable example is their integration as optical signal receivers in laser microphone systems [90]. NIR OPDs have significant potential in secure optical communication technologies for military and industrial applications. By incorporating NIR-OPDs into a laser microphone system, real-time audio transmission was successfully achieved. The circuit diagram of the laser microphone system and the corresponding experimental setup are shown in Figure 10a–c.

When an 808 nm laser beam was directly applied to the NIR-OPD in the receiver unit, an immediate and continuous audio signal was generated, confirming the feasibility of NIR laser-based communication. Furthermore, when transmitting low- and high-frequency audio files separately, the NIR OPD reproduced the signals with minimal distortion, as shown in Figure 10d,e. These results demonstrate the device’s capability to effectively recover audio signals with high fidelity.

**Figure 11:**
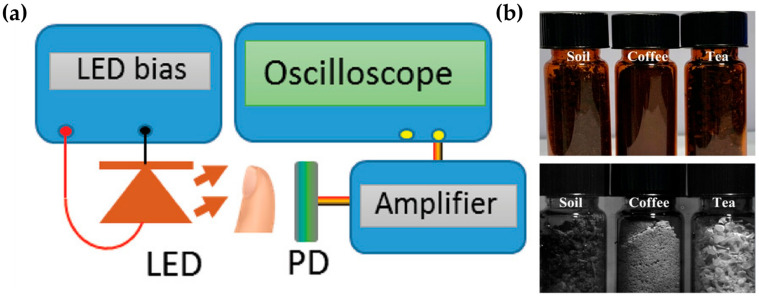
(**a**) Application of a SWIR photodetector: photoplethysmography (PPG) sensor for real-time heart rate monitoring. (**b**) Monitoring application of a NIR photodetector integrated into a smartphone-based system. Top: photographic images of soil, coffee, and tea samples. Bottom: corresponding NIR-detected grayscale images. Panel (**a**) reproduced with permission from Ref. [95]. Copyright 2020, WILEY-VCH Verlag GmbH & Co. KGaA, Weinheim, Germany. Panel (**b**) reproduced with permission from Ref. [97]. Copyright 2023, American Chemical Society.

Another emerging application of OPDs is in real-time monitoring systems. These can be categorized into SWIR-based and NIR-based monitoring approaches [91,92,93,94]. A SWIR-OPD has been utilized in a portable photoplethysmography (PPG) sensor for real-time heart rate monitoring (Figure 11a) [95]. As previously discussed, SWIR light is biocompatible and exhibits deeper tissue penetration compared to NIR light [96]. Therefore, by illuminating regions such as the fingertip or earlobe with SWIR light and monitoring changes in signal intensity, vital information such as heart rate and blood oxygen saturation can be extracted.

For NIR-OPD-based monitoring [97], a NIR photodetector was integrated into a semiconductor circuit and connected to a smartphone camera. When this setup was used to monitor semi-transparent brown glass bottles, information embedded within each bottle could be successfully retrieved (Figure 11b). Although such packaging is difficult to analyze visually, NIR-based monitoring enabled the acquisition of high-quality images revealing internal content and structure.

**Figure 12:**
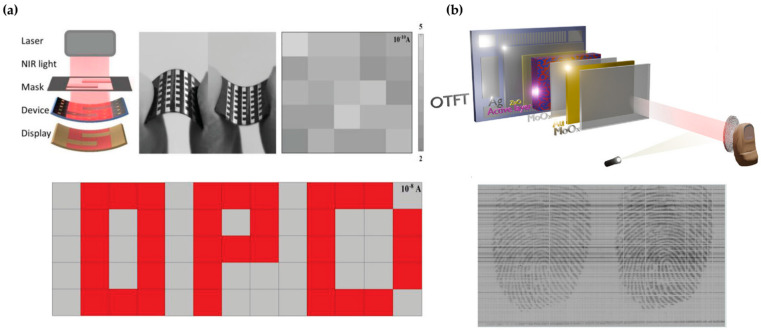
(**a**) Flexible OPD image sensing system and imaging results under 850 nm illumination with patterned letters “O”, “P”, and “D”. (**b**) Application of OPD arrays integrated with OTFTs for fingerprint recognition, with the schematic illustration of the detection system and the corresponding captured fingerprint images. Panel (**a**) reproduced with permission from Ref. [98]. Copyright 2023, Wiley-VCH GmbH. Panel (**b**) reproduced with permission from Ref. [99]. Copyright 2025, Wiley-VCH GmbH.

OPDs can also be employed in image sensing technologies. As shown in Figure 12a, three separate images were captured by projecting light through masks containing different characters [98]. The shapes of the characters were clearly distinguishable from the background due to high contrast, demonstrating the imaging capability of OPDs. These devices are compatible with flexible substrates and exhibit uniform dark current distribution, thereby highlighting their promise for next-generation optoelectronic applications.

Furthermore, OPDs have been applied to fingerprint recognition, where integration with organic thin-film transistor arrays enabled the acquisition of clear digital fingerprint images with distinct ridge and valley patterns (Figure 12b) [99]. These results demonstrate the applicability of OPD-based image sensors for biometric authentication and secure electronics, as well as their potential extension into wearable and portable devices.

## 5. Conclusions and Perspectives

This review has summarized recent progress in near-infrared organic photodetectors from the perspectives of material design and device engineering. Advances in small-molecule and polymer semiconductors have demonstrated that molecular structure control, morphology optimization, and device architecture engineering can significantly improve key performance metrics, including detectivity, photoresponse speed, and spectral coverage in the NIR region.

Despite these achievements, the practical applications of OPDs remain limited. Compared to inorganic photodetectors, OPDs still face challenges in overall performance, long-term operational stability, and environmental robustness, particularly under exposure to moisture and oxygen. In addition, current OPD research is largely driven by academic studies, with relatively limited industrial participation, which has slowed the transition from laboratory demonstrations to commercial products.

Nevertheless, the growing demand for flexible, transparent, and wearable electronic systems highlights application areas where OPDs can offer clear advantages that are difficult to achieve with conventional inorganic technologies. Future research should therefore focus on extending spectral sensitivity toward longer wavelengths, simplifying synthetic routes for low-bandgap materials, improving device stability and lifetime, and developing scalable fabrication processes. A deeper understanding of charge generation, transport, and noise mechanisms, combined with performance-oriented device optimization, will be essential for accelerating the industrialization of OPDs. With continued progress, near-infrared organic photodetectors are expected to emerge as key components in next-generation photonic technologies and flexible optoelectronic systems.

## Data Availability

No new data were created in this study. Data sharing is not applicable to this article.
